# Processing Probability Information in Nonnumerical Settings – Teachers’ Bayesian and Non-bayesian Strategies During Diagnostic Judgment

**DOI:** 10.3389/fpsyg.2020.00678

**Published:** 2020-07-03

**Authors:** Timo Leuders, Katharina Loibl

**Affiliations:** ^1^Institute of Mathematics Education, University of Education, Freiburg, Germany; ^2^Institute of Education, University of Education, Freiburg, Germany

**Keywords:** Bayesian reasoning strategies, information processing, judgment under uncertainty, teachers’ diagnostic judgment, visualization of Bayesian update

## Abstract

A diagnostic judgment of a teacher can be seen as an inference from manifest observable evidence on a student’s behavior to his or her latent traits. This can be described by a Bayesian model of inference: The teacher starts from a set of assumptions on the student (hypotheses), with subjective probabilities for each hypothesis (priors). Subsequently, he or she uses observed evidence (students’ responses to tasks) and knowledge on conditional probabilities of this evidence (likelihoods) to revise these assumptions. Many systematic deviations from this model (biases, e.g., *base-rate neglect*, *inverse fallacy*) are reported in the literature on Bayesian reasoning. In a teacher’s situation, the information (hypotheses, priors, likelihoods) is usually not explicitly represented numerically (as in most research on Bayesian reasoning) but only by qualitative estimations in the mind of the teacher. In our study, we ask to which extent individuals (approximately) apply a rational Bayesian strategy or resort to other biased strategies of processing information for their diagnostic judgments. We explicitly pose this question with respect to nonnumerical settings. To investigate this question, we developed a scenario that visually displays all relevant information (hypotheses, priors, likelihoods) in a graphically displayed hypothesis space (called “hypothegon”)–without recurring to numerical representations or mathematical procedures. Forty-two preservice teachers were asked to judge the plausibility of different misconceptions of six students based on their responses to decimal comparison tasks (e.g., 3.39 > 3.4). Applying a Bayesian classification procedure, we identified three updating strategies: a *Bayesian update strategy* (BUS, processing all probabilities), a *combined evidence strategy* (CES, ignoring the prior probabilities but including all likelihoods), and a *single evidence strategy* (SES, only using the likelihood of the most probable hypothesis). In study 1, an instruction on the relevance of using all probabilities (priors and likelihoods) only weakly increased the processing of more information. In study 2, we found strong evidence that a visual explication of the prior–likelihood interaction led to an increase in processing the interaction of all relevant information. These results show that the phenomena found in general research on Bayesian reasoning in numerical settings extend to diagnostic judgments in nonnumerical settings.

## Introduction

Judgments on other people’s knowledge, even when based on accurate knowledge and sound evidence, are uncertain and fallible (Nickerson, 1999). For example, when teachers assess students’ abilities, their diagnostic judgments are based on evidence available in a concrete situation (e.g., the student’s solution on a task) and on their prior knowledge on the student’s abilities. Generally, teachers’ judgments are framed by their theoretical knowledge (e.g., pedagogical content knowledge about typical misconceptions) (Schrader, 2009; Herppich et al., 2018; Loibl et al., 2020).

Often, such diagnostic judgments are investigated with respect to their accuracy and their dependence on personal and situational characteristics (for a meta-analysis, see Südkamp et al., 2012). Less often to be found is research on the cognitive processes underlying the diagnostic judgments of teachers (e.g., Glock and Krolak-Schwerdt, 2014; Pit-ten Cate et al., 2016). For many years, diagnostic judgments of clinicians have been investigated with a focus on cognition, e.g., within the heuristics-and-bias paradigm (cf. Round, 2001; Gill et al., 2005; Croskerry, 2009) and with respect to Bayesian reasoning (Edwards, 1968; Gigerenzer and Hoffrage, 1995; Griffiths et al., 2008).

A diagnostic judgment of a teacher can be seen as an inference from manifest observable evidence on a student’s behavior to his or her thinking or latent traits. Usually, such an inference is inherently uncertain. Hence, the result of a diagnostic judgment is rather a set of hypotheses about the observed student with varying plausibility than an unequivocal classification of the student. For example, a student may give a wrong answer when asked to compare two decimals – e.g., stating that 4.8 < 4.63 – because he or she treats the fractional parts of decimal numbers as natural numbers (8 < 63). Many students do so consistently (Moloney and Stacey, 1997) with a high probability. However, an uncertainty remains, since even students with this misconception may occasionally solve a task correctly. In addition, students with other misconceptions may give the same wrong answer (e.g., by ignoring the decimal point: 48 < 463), and even those students who do understand decimals well may occasionally (i.e., with a low probability) give a wrong answer. Therefore, the inference from the observed behavior to an underlying cognition is uncertain, even though the students’ cognitions are well known, as is the case for comparing decimals.

From the perspective of the *accuracy of teachers’ judgments*, these uncertainties can be interpreted as reduced diagnosticity either due to imperfect specificity or sensitivity of the tasks or due to inadequate knowledge or reasoning of the teachers. As a consequence, one would strive to optimize the tasks or to train the teachers. However, from the perspective of the *cognitive processes underlying the judgment*, one may probe deeper into the teachers’ thinking and ask how teachers incorporate such uncertainties in their judgments.

A prominent approach that describes judgments under conditions of uncertainty is the Bayesian model of inference (Edwards, 1968; Gigerenzer and Hoffrage, 1995; Cosmides and Tooby, 1996; Griffiths et al., 2008): An initial uncertainty is modeled as a set of assumptions (hypotheses) about a situation, with subjective probabilities for each hypothesis (often called “priors” or “base rates”). Subsequently, observed data (i.e., “evidence”) is used to update these probability assumptions – provided one knows the plausibility of the evidence, expressed by its conditional probabilities (also called “likelihoods”).

The ideal probabilistic model for this “updating process” is given by formal Bayesian reasoning. The Bayes’ formula can be used to describe, by means of probability calculus, how the probabilities of hypotheses change when evidence is produced:

$$\begin{array}{ccccccc} P\left(H_{i}|E\right) & = & P\left(E|H_{i}\right) & \times & P\left(H_{i}\right) & \times & \frac{1}{{\text{Σ}}_{j}P(E|H_{j})P(H_{j})}\\ \begin{array}{c} \text{posterior}\\ \text{probability}\\ \text{of hypothesis }H_{i},\\ \text{given data }E \end{array} & & \begin{array}{l} \text{likelihood of}\\ \text{data }E\text{ under}\\ \text{hypothesis }H_{i} \end{array} & & \begin{array}{c} \text{prior}\\ \text{probability of}\\ \text{hypothesis }H_{i} \end{array} & & \begin{array}{l} \text{Normalization}\\ \text{to have sum of}\\ \text{probabilities = 1} \end{array} \end{array}$$

Many researchers argue that people are capable of intuitively applying the Bayesian update strategy, represented numerically by this formula, when they make judgments under conditions of uncertainty (e.g., Martins, 2006; Zhu and Gigerenzer, 2006; Girotto and Gonzales, 2008). However, there is also much evidence for systematic deviation from this model. Some of the most often reported biases relate to disregarding the prior distribution (*base-rate neglect*, Kahneman and Tversky, 1996, p. 584) by only considering the likelihoods proportionally: *P*(*H*_*i*_|*E*)∝*P*(*E*|*H*_*i*_) – in an extreme form even mistaking one conditional probability for the other: *P*(*H*_*i*_|*E*) = *P*(*E*|*H*_*i*_) (*inverse fallacy*, Villejoubert and Mandel, 2002). Another biased strategy would be to assume wrong base rates for the hypotheses *P*(*H*_*i*_), for example an *anchoring bias* caused by an expert blind spot, i.e., experts’ tend to overestimate the knowledge of novices (Nathan and Koedinger, 2000). We use the term Bayesian (update) strategy only for the (approximative) application of the Bayes’ rule above. However, it might be sensible to apply a broader understanding of Bayesian reasoning (Baratgin and Politzer, 2010; Mandel, 2014; see section “Discussion”).

In the context of diagnostic judgments of teachers, the diagnostic situation is structurally analogous to the judgment situations indicated in the literature above, which does not refer to teachers: A teacher’s prior assumptions (hypotheses) on a students’ latent trait (e.g., a decimal-comparison misconception) relies on his or her estimation of the typical prevalence (base rates) of these misconceptions. A student’s behavior or response to a task (manifest data, evidence) can be used to revise these assumptions (by updating the prior hypotheses).

**FIGURE 1:**
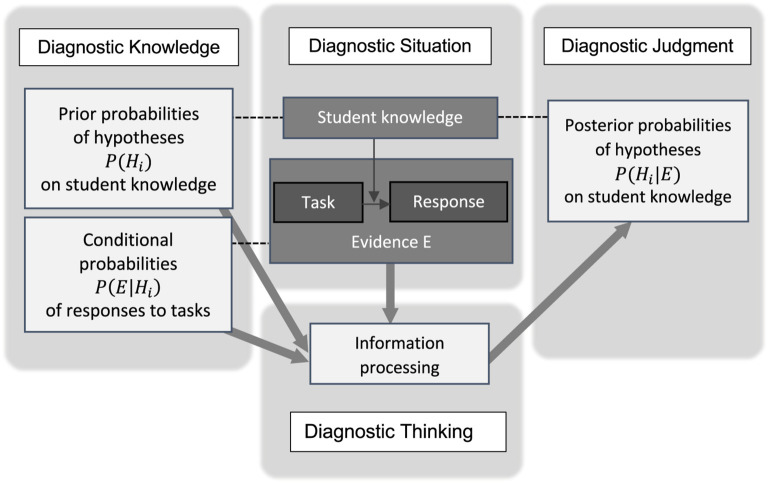
The structure of teacher’s diagnostic judgment based on knowledge, evidence, and information processing and the role of uncertainty.

The structure of this updating process in the context of teachers’ diagnostic judgment on student knowledge is displayed in Figure 1: In order to update the probabilities of the hypotheses [from *P*(*H*_*i*_) to *P*(*H*_*i*_|*E*)], the teacher processes his or her diagnostic knowledge (i.e., prior probabilities and conditional probabilities) as well as the information provided in the diagnostic situation (i.e., the evidence). Uncertainty plays a major role in this updating process: Students do not respond consistently (cf. conditional probabilities), and different student knowledge may lead to same responses (ambiguity/limited diagnosticity).

However, a teacher’s situation also differs from the situation typically encountered in research on Bayesian reasoning, since these pieces of knowledge and information are usually not explicitly represented by numbers but only by qualitative and subjective estimations in the mind of the teacher. Any assumed process of Bayesian reasoning therefore also relies on processing such information in a qualitative, nonnumerical way.

Against this background, we ask to which extent individuals, who are asked for a diagnostic judgment in a situation as described here, are able to (approximately) apply a rational Bayesian strategy or resort to other “biased” strategies of processing information for their diagnostic judgments. We explicitly pose this question with respect to nonnumerical settings, bearing in mind that Bayesian and other types of reasoning are already researched and reported extensively for numerical settings.

To systematically investigate this question, we develop a rich scenario of diagnostic judgment (three possible hypotheses, diagnostic tasks with limited reliability, and diagnosticity) that is displayed in an optimized way for accessing all relevant information (prior probabilities, conditional probabilities, updating procedure) in a qualitative way, without recurring to numerical representations or mathematical procedures (as, e.g., systematically investigated in Hoffrage et al., 2015).

## Theoretical Background

### Teachers’ Diagnostic Judgments Under Uncertainty – Through the Lens of Bayesian Reasoning

Identifying learners’ misconceptions is one key task of teachers in order to address these misconceptions adequately in teaching (Weinert et al., 1990). However, such diagnostic judgments are far from straightforward and – like many types of human judgment – characterized by uncertainty (Tversky and Kahneman, 1974; Kozyreva and Hertwig, 2019; Mandel et al., 2019). As described earlier, students with different misconceptions can show the same behavior (i.e., give an identical answer to a task) – either because the task cannot distinguish between several misconceptions or because the students do not respond consistently. Both phenomena are sources of uncertainty for teachers’ diagnostic judgments. In order to judge in a rational way, teachers have to apply effective strategies to deal with the diverse uncertainties. When doing so, teachers usually do not resort to numerical or mathematical procedures of probability calculus but take into account their knowledge (gained by experience or based on literature) on the assumed relative probabilities of the misconceptions and the expected (in)consistency of students’ answers in a qualitative, nonnumerical manner. In other words, they may engage in Bayesian reasoning without applying the explicit Bayesian formula (cf. Martins, 2006). Although the literature on Bayesian reasoning in many different contexts abounds, all studies rely on numerical representation and calculation of some sort, and no research relates to the situation of teachers’ diagnostic judgments as depicted in Figure 1. Still, the literature on Bayesian reasoning provides many insights into various strategies and biases in Bayesian reasoning and viable support structures to influence these strategies systematically, as outlined in the following.

There is evidence that humans are capable of utilizing Bayesian update strategies when making judgments under uncertainty (Martins, 2006; Girotto and Gonzales, 2008). Even children are able to do so, at least if the information is provided in natural frequencies instead of probabilities (Zhu and Gigerenzer, 2006; Pighin et al., 2017). However, as indicated above, children and adults also often fail to apply the Bayesian update strategy (e.g., Gigerenzer and Hoffrage, 1995; Weber et al., 2018). Instead, they consistently process only a part of the relevant information, resulting in reasoning strategies that deviate from optimal Bayesian reasoning (e.g., Gigerenzer and Hoffrage, 1995; Zhu and Gigerenzer, 2006; Cohen and Staub, 2015).

There is some discussion whether it is appropriate to consider these strategies defective (using the term “biased”) or whether they may be effective in certain situations (ecological rationality: Simon, 1955; Gigerenzer and Hoffrage, 1995). However, this discussion is not relevant for our investigation, since we do not address the questions of effectiveness (i.e., ecological validity) of the strategies under investigation.

Against this background, two questions and the respective lines of research (although not conducted specifically for the case of teachers’ diagnostic judgments) are of relevance for our research interest:

(1) Which (biased) strategies of processing (nonnumerical) information do individuals apply, when *not* following a Bayesian update strategy?
(2) How can individuals be supported in (approximatively) applying an Bayesian update strategy?

#### Biased Strategies of Processing Information for Updating Judgments

One of the most familiar and often studied judgment situations refers to a medical test of an illness with given prevalence [i.e., base rate *P*(*H*)], a given sensitivity [i.e., positive-when-true rate, likelihood *P*(*E*|*H*)] and a given specificity [i.e., negative-when-false rate *P*(¬*E*|¬*H*)] (e.g., Gigerenzer and Hoffrage, 1995). In such a situation, the probability that a person, selected at random, who receives a positive test result actually has the disease *P*(*H*|*E*) can be calculated according to the Bayes rule. The posterior probability *P*(*H*|*E*) is the rational choice for the judgment on the patient’s state given the evidence of the test. Since the base rate is low in most medical diagnostic test situations, the Bayes rule leads to a much lower posterior probability estimations than most individuals typically estimate (ibid.), even when strongly supported (Weber et al., 2018). Indeed, research has shown that humans often do not apply the Bayes rule, resulting in biased judgments, where the most often reported biases in judgment updating relate to disregarding the prior distribution (base-rate neglect, Kahneman and Tversky, 1996, S. 584).

In a systematic analysis on the types of update strategies in the context of Bayes reasoning tasks (i.e., tasks with a similar structure to the prototype described above), Cohen and Staub (2015) showed that most participants’ judgment strategies amount to not making use of all sources of information (prior probabilities of hypotheses and likelihoods of evidence under each hypothesis), leading to biased update strategies. They further provided evidence that most participants seem to estimate the posterior probability based on only one of the multiple provided probabilities or by computing a weighted sum of several, but not all probabilities. In their studies, the most frequently used pieces of information were the likelihood of the evidence (i.e., positive-when-true rate) and the likelihoods of the evidence under the other hypotheses (i.e., positive-when-false rate).

The findings of Cohen and Staub (2015) rely on an analysis of intraindividual consistency in strategy use. Thereby, they substantiate the earlier classification of interindividual differences in strategies by Zhu and Gigerenzer (2006): In their studies with fourth to sixth graders and adults, they also found strategies focusing on one probability. Subjects either considered only the priors *P*(*H*) (called *conservatism*, Edwards, 1968; or *base rate only*, Gigerenzer and Hoffrage, 1995) or only the likelihood of the evidence at hand *P*(*E*|*H*) (called *representative thinking* or *Fisherian*, Gigerenzer and Hoffrage, 1995; *inverse fallacy*, Villejoubert and Mandel, 2002). In their studies, no one used the *joint occurrence* of the evidence (*P*(*E*|*H*)⋅*P*(*H*) = *P*(*E*∧*H*)), a strategy found by Gigerenzer and Hoffrage (1995). Subjects who actually computed a weighted sum focused only on the evidence [e.g., *P*(*E*|*H*)/Σ*P*(*E*|*H*_*i*_)], called *evidence only* (Zhu and Gigerenzer, 2006). These subjects took the likelihoods of the evidence under all hypotheses into account (i.e., true and false positive rate) but disregarded the base rate. Thus, this strategy can also be considered a type of base-rate neglect (Tversky and Kahneman, 1974; Bar-Hillel, 1983). Gigerenzer and Hoffrage (1995) found another similar strategy (*likelihood subtraction*), in which subjects take into account more than a single likelihood in their computation in a subtractive fashion and ignore the base rate [*P*(*E*|*H*)−*P*(¬*E*|¬*H*)]. Zhu and Gigerenzer (2006) found an additional strategy, not reported elsewhere, which they called “Pre-Bayes.” It corresponds to taking the correct denominator but focusing on the positive-when-true rate as numerator. While the children in their study frequently used this strategy, it may have been triggered by the presentation of the Bayes problems with natural frequencies, which makes the positive-when-true rate salient. Table 1 provides an overview of the most common strategies. From the point of view of information processing, they can be categorized as prior-only strategies (POS), single evidence strategies (SES), combined evidence strategies (CES), and the Bayesian update strategy (BUS).

**TABLE 1:** Overview of most common update strategies.

		Processed information		
Strategy types, variants/denotations		Likelihood/positive-when-true rate	Likelihoods of alternatives/positive-when-positive rate	Prior probabilities/base rate
Prior-only strategy (POS)	Conservatism (Edwards, 1968; Zhu and Gigerenzer, 2006); base-rate only (Gigerenzer and Hoffrage, 1995)			X
Single evidence strategies (SES)	Representative thinking (Zhu and Gigerenzer, 2006); Fisherian (Gigerenzer and Hoffrage, 1995); inverse fallacy (Villejoubert and Mandel, 2002)	X		
Combined evidence strategies (CES)	Evidence only (Zhu and Gigerenzer, 2006); likelihood subtraction (Gigerenzer and Hoffrage, 1995)	X	X	
Bayesian update strategy (BUS)	Bayesian update (correct application of the Bayes’ rule)	X	X	X

The multitude of erroneous strategies appears to suggest that humans do not succeed well in situations of Bayesian reasoning, even when the situation is presented in an accessible way, using natural frequencies and visual representations (Weber et al., 2018). Nevertheless, Martins (2006) argued that humans do take uncertainties into account by revising their judgments based on new information in a way that resembles the rational Bayesian strategy. Similarly, Nickerson (1999) stated that the refinement of one’s knowledge on people relies on an ongoing adjustment process and is based on evidence that one collects. The facts that Bayesian reasoning has been identified at least for some situations, groups, and cases by prior research (e.g., Gigerenzer and Hoffrage, 1995; Zhu and Gigerenzer, 2006; Cohen and Staub, 2015) and that any form of reduction of numerical calculation and information saliency of presentation appears to be effective (see section “Supporting the Application of the Bayesian Update Strategy”) support the assumption that humans are, in principle, capable of intuitively applying the essence of the Bayes’ rule, depending on the situational conditions.

In a nutshell, the strategies differ in the amount and type of processed information. While research has shown individual differences with regard to the use of the available information (Cohen and Staub, 2015), the perception and processing of information also depend on the representation of the situation and the amount of support, which we will analyze in the next section.

#### Supporting the Application of the Bayesian Update Strategy

How individuals process the relevant information for Bayesian reasoning highly depends on the situation (cf. McDowell and Jacobs, 2017). During the last decades, research has investigated how to represent the information in a way that supports individuals in applying the Bayes update strategy. The common idea is to assist the individuals in gathering the relevant information and constructing an adequate structural mental model of the situation. The most prominent representation strategies that have been shown to be effective are (a) using natural frequencies instead of probabilities (cf. meta-analysis by McDowell and Jacobs, 2017) and (b) visualizations that increase the salience of the structure (e.g., Khan et al., 2015; Böcherer-Linder and Eichler, 2017).

Multiple studies have shown that people are better in solving Bayesian tasks that are represented with natural frequencies (also called natural sampling) than tasks that present the information in the form of probabilities (e.g., Zhu and Gigerenzer, 2006; Hill and Brase, 2012; for a meta-analysis, see McDowell and Jacobs, 2017). The Bayesian update strategy is computationally simpler if probabilities are represented as joint frequencies because the base rate is already contained in the joint frequencies, and, therefore, there is no need to additionally include the base rate in the calculation. However, this advantage is only relevant in settings with numerical representations and calculation demands. In addition to the reduced computational load, it has been argued that, in Bayesian tasks with natural frequencies, the information is given in the same chronological order in which information is naturally acquired (ecological rationality framework, Gigerenzer and Hoffrage, 1995). Moreover, the way the information is provided highlights the structure of the task (i.e., the nested-set relations, Sloman et al., 2003) and thereby facilitates the construction of an adequate situation model.

Another way to increase the salience of the structure of the situation (i.e., nested-set relation) is to provide adequate visualizations (for an overview, see Khan et al., 2015), such as tree diagrams (Yamagishi, 2003; Weber et al., 2018) or unit squares (Böcherer-Linder and Eichler, 2017; Pfannkuch and Budgett, 2017). Notably, visualizations increase the performance not only for tasks presented with probabilities but also for tasks presented with natural frequencies (McDowell and Jacobs, 2017), indicating an added value in additionally presenting the nested-set structure with visualizations. When comparing different visualizations, Böcherer-Linder and Eichler (2017) argue that the tree diagram reveals the nested-set relation only in a numerical way, whereas the unit square adds a geometrical, qualitative representation. This assumption receives support by the finding that the unit square supported the correct application of the Bayes’ rule more than the tree diagram. One can assume that such nonnumerical representations, which render saliency to relevant information (to overall structure and to the relative sizes) support Bayesian reasoning. However, so far, visualizations have only been provided in addition to the numerical values, not in isolation.

Another potential way of supporting the use of the available information would be to highlight the relevance of the information. In a different area of teachers’ diagnostic skills (noticing students’ beliefs), Zeeb et al. (2019) have shown that highlighting the relevance of integrating different types of knowledge (and giving an example) significantly improved the integrated used of different types of knowledge. It seems reasonable that such an instruction on the relevance of integration could also be beneficial in the context of judgment under uncertainty by fostering the use and integration of all available information.

#### Modeling Bayesian Reasoning in Nonnumerical Settings

In our study, the focus on teachers’ diagnostic judgments is accompanied by two central premises for the theoretical framing and the ensuing investigations.

As first premise, we recognize that the literature on Bayesian reasoning focuses – by always providing numerical information – on applying the Bayes rule by (more or less extensive) calculation. While the numerical information is often accompanied with graphical representation to visualize the structure of the situation (e.g., Böcherer-Linder and Eichler, 2017), no study solely relied on qualitative, nonnumerical information. However, in the context of teachers who update their judgments regarding their students’ misconceptions based on the students’ solution, the pieces of information are rather not represented by numbers but only by qualitative estimations, and thus, the process of Bayesian reasoning also relies on processing such information in a qualitative and approximative way.

As a second premise, we note that research explains the fact that humans often fail to apply the Bayesian update strategy appropriately on the basis that they often do not use (perceive and process) all relevant information and instead apply different biased strategies. While such strategies have been found in the context of numerical Bayesian reasoning, it seems reasonable to assume that similar strategies also appear in processing the available qualitative (i.e., nonnumerical) information in the context of judgments under uncertainty. More precisely, the following strategies (known from the literature on numerical Bayesian reasoning) can also be expected in nonnumerical settings, considered here:

(a) the rational (i.e., mathematically correct) BUS, that is, processing the conditional probabilities of a student’s solutions under all plausible hypotheses (likelihoods of evidence) and the prior probabilities of these hypotheses,
(b) a CES (cf. evidence only: Zhu and Gigerenzer, 2006; Likelihood subtraction: Gigerenzer and Hoffrage, 1995), that is, ignoring the prior probabilities, but combining the data likelihoods regarding all hypotheses (by considering a normalized, relative size),
(c) a SES (cf. representative thinking: Zhu and Gigerenzer, 2006; Fisherian: Gigerenzer and Hoffrage, 1995; inverse fallacy: Villejoubert and Mandel, 2002), that is, only considering the data likelihood regarding the most probable hypothesis (i.e., ignoring both the data likelihoods regarding the alternative (less likely) hypotheses and the prior probabilities).

However, a POS (cf. conservatism: Edwards, 1968; Zhu and Gigerenzer, 2006; base rate only: Gigerenzer and Hoffrage, 1995), that is, not updating the judgment at all, seems less likely as teachers generally focus on and react to their students’ responses and, thereby, naturally process the evidence.

Since we are interested in the use of information rather than the mere perception, we aim at constructing a situation in which all information necessary for the individual to generate a judgment is available and maximally salient. We then investigate whether individuals under these circumstances actually perform judgments that resemble Bayesian reasoning. To specify a scenario for our investigation, we first describe the types of hypotheses and evidence on students that we restrict our investigation to (see section “Decimal Strategies and their Diagnostics”) and then specify the environment (diagnostic situation) which frames the judgments processes of the participants (see section “A Computer-Based Setting for Nonnumerical Diagnostic Strategies”).

### Decimal Strategies and Their Diagnostics

In order to investigate the expected updating strategies described above in a single coherent framework of teachers’ diagnostic strategies, we use the case of diagnostic judgment on students’ decimal comparison misconceptions, since in this area, a theory on students’ (mis)conceptions is empirically well founded (e.g., Moloney and Stacey, 1997).

Although these misconceptions are sometimes called strategies, in the following, we prefer using the term misconceptions to reduce confusion with the strategies applied by teachers during the diagnostic judgment process.

The three most prevalent decimal-comparing misconceptions are shown in Table 2. The table also presents examples for the most frequent types of diagnostic tasks to detect the misconceptions.

**TABLE 2:** Common misconceptions when comparing decimal fractions (cf. Moloney and Stacey, 1997).

**Decimal comparing misconceptions**	**Description**	**Diagnostic task and response**
Whole-number misconception (WN)	Students interpret the decimal point as a separator of two numbers and consider the sizes separately	“4.125 > 4.7 because 125 > 7”
Ignore-decimal-point misconception (ID)	Students ignore the decimal point and proceed as if they compared natural numbers	“2.45 < 1.328 because 245 < 1328”
Shorter-is-larger misconception (SL)	Some students consistently choose the number with fewer decimal places as the larger	“2.3 > 2.67 because tenths are larger than hundredths” or “because a third is larger than 1/67”

Studies on the prevalence of these misconceptions often investigate students from different age groups, countries, and school types (Sackur-Grisvard and Léonard, 1985; Nesher and Peled, 1986; Padberg, 1989; Resnick et al., 1989; Moloney and Stacey, 1997; Steinle, 2004; Heckmann, 2006). They reveal that there is a considerable variation depending on the stage of curriculum. For example, the whole-number misconception is dominant in younger children. The shorter-is-larger-conception typically arises after the introduction of fractions and then decreases with each grade. In Germany, at the start of grade 5 (before the introduction of fractions), a relative frequency of the misconceptions WN/ID/SL of 60%:30%:10% (Heckmann, 2006) is a plausible assumption for a distribution of misconceptions and will be used in our study.

### A Computer-Based Setting for Nonnumerical Diagnostic Strategies

In section “Teachers’ Diagnostic Judgments Under Uncertainty – Through the Lens of Bayesian Reasoning,” we obtained an overview on Bayesian judgment in order to generate plausible assumptions on teachers’ information processing strategies during diagnostic judgments. In section “Decimal Strategies and Their Diagnostics,” we analyzed a content area (comparing decimals) in order to define a research-based knowledge base on students’ misconceptions, diagnostic tasks, and the uncertainties connected to this topic, initially independently from the teacher using this knowledge.

In order to investigate the genesis of diagnostic judgments (a) under the condition of uncertainty and (b) in a nonnumerical setting, we use this theoretical basis to follow the research strategy of the DiaCoM framework (Loibl et al., 2020), which was designed to generally structure research on diagnostic judgment processes. Its components are the following: (1) specification and systematic variation of the diagnostic situation with regard to perceptible information (here: evidence on students’ solutions to given tasks), (2) specification of relevant diagnostic knowledge (here: prior probabilities and conditional probabilities), (3) specification of diagnostic thinking as cognitive processing of information and knowledge (here: the use of information during Bayesian or non-Bayesian updating), and (4) operationalization of diagnostic judgment (here: posterior probabilities) and prediction of this judgment.

(1) Specification of the Diagnostic Situation

Identifying students’ misconceptions is one key task of teachers in order to address these misconceptions adequately. However, these judgments regarding students’ misconceptions often are not straightforward. As described earlier, students with different misconceptions can come to the very same answer – either because the task cannot distinguish between several misconceptions or because the students do not follow their erroneous strategy with complete consistency. Both factors lead to judgments under uncertainty.

In our study, the students’ misconception space is restricted to the three most frequent decimal comparing misconceptions as described above (see section “Decimal Strategies and their Diagnostics”). This restriction also implies that we do not include students who fully understand decimals and therefore solve all comparison tasks correctly (most of the time). Thus, a teacher in our study assumes to encounter a student who pertains to one of three mutually exclusive misconception groups. This defines the set of three hypotheses (WN, ID, and SL) for the diagnostic judgment.

A piece of evidence that a teacher encounters in our study consists of a student’s response to one of the three diagnostic tasks as presented in Table 3. Each task is assumed to have a sensitivity of 80% throughout all cases. We keep this feature of the diagnostic tasks constant because, in this study, we are not interested in the influence of variation in sensitivity but in the use or disregard of information on evidence in general. Furthermore, assuming the same sensitivity for all tasks reduces the amount of diagnostic information that has to be processed.

**TABLE 3:** Pattern of most likely response (evidence *E*) of each task under the condition of a student’s misconception (hypothesis *H*).

Hypothesis	Decimal comparing misconceptions	Task 1: 4.8 > 4.63	Task 2: 3.7 > 3.02	Task 3: 3.49 > 3.4
H1	Whole-number misconception (WN)	Wrong	Right	Right
H2	Ignore-decimal-point misconception (ID)	Wrong	Wrong	Right
H3	Shorter-is-larger misconception (SL)	Right	Right	Wrong

*The likelihood of the evidence indicated in the table *isP*(*E*|*H*) = 0.8.*

A feature that typically arises in diagnostic judgments is the phenomenon that the tasks do not detect students’ misconceptions unambiguously – a situation that has been only rarely addressed in research on Bayesian reasoning. The resulting pattern in the set of evidences (three task types with two responses depending on three misconceptions) used in this study is presented in Table 3. It results from the combination of the (erroneous) mathematical student reasoning pertaining to each misconception and the mathematical structure of the numbers in the task. An in-depth analysis of all conceivable task types to induce erroneous results and detect misconceptions (i.e., varying length of the part before and after comma, position of zeroes, especially leading and trailing zeroes) showed that the task types chosen here are most straightforward to allow diagnosing the misconceptions. Another task type, not used here, would be, e.g., 3.95 > 3.76, which would not allow to differentiate between any two of the misconceptions.

The evidence presented in a single diagnostic situation comprises a diagnostic task and a student’s response, one at a time. To each teacher, several cases of different students are presented in a row.

(2) Specification of Diagnostic Knowledge

**FIGURE 2:**
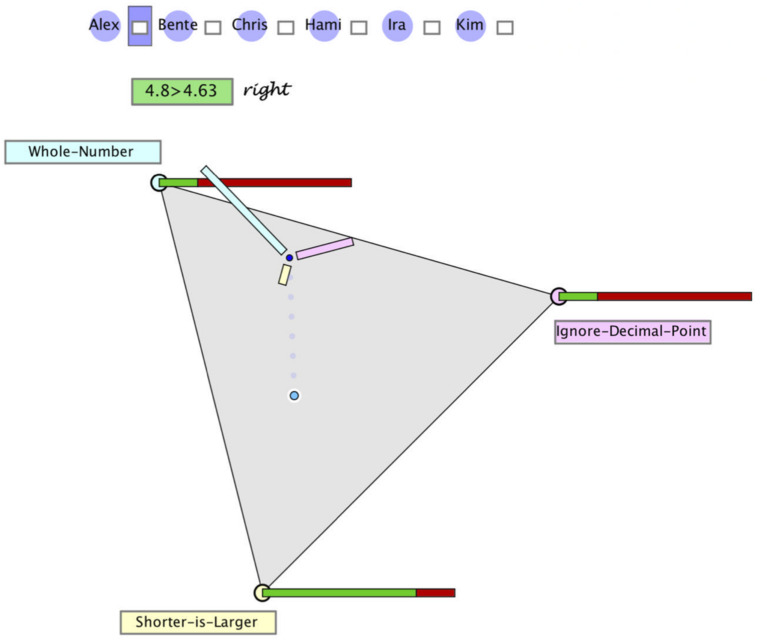
Hypothegon representing a ternary hypothesis space, a judgment as a position in this space, and the evidence likelihoods (conditional probabilities) of the response. In order to make a judgment (posterior), one can drag the point to a new position.

In order to achieve adequate judgments (probabilities for possible hypotheses), an individual has to take into account diagnostic knowledge on different probabilities: the prior probabilities for the different misconceptions as well as the likelihoods for each misconception given certain evidence. Figure 2 illustrates how this information can be displayed graphically in a distinct and comprehensive manner.

**FIGURE 3:**
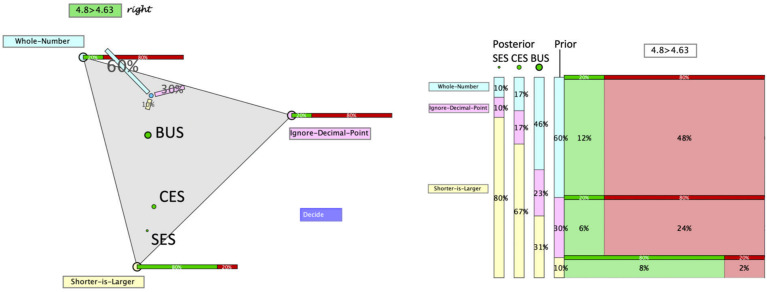
Hypothegon (left hand side) and visualization of update strategies (right hand side).

• The three hypotheses (WN, DL, SL) are represented as vertices spanning a planar equilateral triangle (see Figure 3).

• The interior and boundary of this triangle comprises all possible distributions of three probabilities: (*p*_1_, *p*_2_, *p*_3_) with *p*_1_ + *p*_2_ + *p*_3_ = 1, and thus constitutes a ternary hypothesis space (or for short “hypothegon”).^1^ A location at a vertex indicates the certainty of the hypothesis (e.g., *p*_1_ = 1); the center point represents a uniform distribution (*p*_1_ = *p*_2_ = *p*_3_ = 1/3).
• The prior distribution is represented twofold: with the position of the “prior point,” the prior distribution and by the length of three bars, pointing to the respective hypotheses. Figure 2 shows the position and bar diagram for a prior probability (base rate) of 60, 30, and 10% of the three misconceptions.
• The likelihoods of the two possible responses (right/wrong) to a given task are represented qualitatively as stacked bars at the corners of the hypothegon. For example, the task 4.8 > 4.63 is responded correctly. The likelihood of a correct response by a student with misconception WN is 20%, same as by a student with misconception ID. The likelihood of a correct response by a student with misconception SL is 80%.

To be able to process the given information, teachers require knowledge on the misconceptions (cf. see section “Decimal Strategies and Their Diagnostics”), and they have to understand the meaning of the probabilities involved (cf. see section “Teachers’ Diagnostic Judgments Under Uncertainty – Through the Lens of Bayesian Reasoning” and *Specification of the Diagnostic Situation*). Both types of knowledge can be manipulated by instruction. Furthermore, teachers have to pay attention to all information given. As indicated in section “Supporting the Application of the Bayesian Update Strategy,” this attention can be manipulated by the representation of the information (especially the nested-set relation) or by relevance instruction.

(3) Operationalization of Observable Diagnostic Judgment

In the same manner in which the prior probabilities for hypotheses are located in the hypothegon, also the updated hypotheses, i.e., the posterior probabilities, can be represented as locus within the hypothegon, and the updating process amounts to moving the point to a new position. The new locus of the point represents the qualitative estimation of the posterior probabilities. In this way, the updating procedure can be executed in an intuitive manner: moving closer towards a hypothesis expresses a strengthened belief, positioning the point between two hypotheses expresses (subjective) ambiguity (see Figure 2).

(4) Specification of Diagnostic Thinking

With diagnostic thinking, Loibl et al. (2020) refer to the assumed processing of information. As summarized in section “Modeling Bayesian Reasoning in Nonnumerical Settings,” we assume that teachers process all or only part of the information given (i.e., evidence, prior probabilities, and/or likelihoods), corresponding to the update strategies discussed in section “Teachers’ Diagnostic Judgments Under Uncertainty – Through the Lens of Bayesian Reasoning.” Although teachers are not assumed to mathematically calculate the posterior probabilities, the four update strategies can still be presented by formulas. The formulas as well as the results of the three update strategies for the example given above are displayed at the right side of Figure 3. The fourth strategy (prior only) is excluded from our analysis, since it would be realized by *not* moving the point – which is an improbable behavior under the circumstances of the study. The green dots in the triangle in Figure 3 correspond to the locus of the point for the posterior probabilities, when teachers judge according to one of the three strategies:

(a) They may only process the likelihood of the hypothesis with the highest likelihood (SES). In the example, this is SL with a likelihood of 80%. When no further information is processed, this likelihood is taken as probability of the hypothesis. We assume that the remaining probability of 20% is (possibly implicitly) distributed over the remaining hypotheses. This strategy leads to the locus of the smallest green dot.
(b) They may process and balance the likelihoods of all three hypotheses (CES), i.e., they consider the following values: WN 20%, ID 20%, and SL 80%. The relative sizes (i.e., normalized to give a sum of 1) are taken as probabilities of the hypotheses. This would result in WN 17%, ID 17%, and SL 67%. These posterior probabilities are represented by the locus of the middle green dot.
(c) They may process all relevant information following the Bayes’ rule (BUS), which leads to the following posterior probabilities: WN 46%, ID 23%, and SL 31%, represented by the locus of the biggest green dot.

## Research Questions

When people update their hypotheses based on uncertain evidence (e.g., teachers’ updating their assumptions based on students’ solutions), they may only have access to nonnumerical information. When only part of the information on relevant probabilities is processed, this may result in updating strategies different from Bayesian reasoning. We investigate the following research question (RQ1):

Can common types of updating strategies known from numerical settings also be detected in a nonnumerical setting?

H1: We hypothesize that the following strategies are identifiable within the nonnumerical setting described above:

• a *Bayesian update strategy* (BUS), that is, processing all probabilities (priors and likelihoods),
• a *combined evidence strategy* (CES), that is, ignoring the prior probabilities (also known as base rate neglect), but taking into account the likelihoods of the evidence under all hypotheses,
• a *single evidence strategy* (SES), that is, ignoring the prior probabilities (base-rate neglect) and only using the likelihood of the most probable hypothesis (also known as inverse fallacy).

In our setting, the nonnumerical information on the probabilities relevant for Bayesian reasoning is represented in a salient manner. However, the existence of non-Bayesian updating strategies within this setting (as commonly found in other settings, see above) suggests that not all individuals use all of this information. In numerical settings, this can be influenced by means of instruction or representation. Therefore, we investigate the following research question (RQ2):

Does instruction on the relevance of using all probabilities (priors and likelihoods) increases the processing of more information represented in the nonnumerical setting?

H2: We hypothesize that the instruction increases the individuals’ processing of information, leading to an increase in the BUS and a decrease in the SES.

## Methods

### Participants

The 26 preservice teachers who participated in the study all completed their bachelor in teaching mathematics and took courses in a master program on teaching mathematics at the time of the study. Participants were randomly assigned to two conditions: one condition with a salient presentation of priors and likelihoods (“control condition”) and one condition with an additional instruction on the relevance of priors and likelihoods (“relevance instruction condition”, see section “Influence of instruction (RQ2)”]. With these conditions, we aim to increase the variance of the different strategies in order to identify strategy types (RQ1) and to test our assumptions regarding the processing of information (RQ2). The descriptive statistics of the participants are presented in Table 4.

**TABLE 4:** Descriptive statistics of participants of study 1 and study 2 [means (SD)].

	Study 1			Study 2
	(1) Control condition	(2) Relevance instruction condition	Total (1 + 2)	Interaction explication condition
*N*	14	12	26	16
Gender female/male	10/4	7/5	17/9	9/7
Age	24.14 (1.66)	23.25 (1.14)	23.73 (1.48)	24.06 (1.61)
Semester	7.93 (1.14)	7.12 (0.58)	7.58 (0.99)	8.00 (1.32)
High school diploma, grade 1(best) through 5	2.51 (0.43)	2.31 (0.42)	2.42 (0.43)	2.33 (0.52)
Understanding of setting (max. 3 points)	3.00 (0.00)	2.92 (0.29)	2.96 (0.20)	2.90 (0.30)

### Generating Evidence on Updating Strategies (RQ1) – The Nonnumerical Setting

In our study, the investigation of Bayesian reasoning in nonnumerical settings is framed by a scenario of diagnostic judgment as described in section “A Computer-Based Setting for Nonnumerical Diagnostic Strategies.” It is a complex judgment situation with

• three possible hypotheses (on students’ misconceptions),
• two possible outcomes (right/wrong responses),
• three task types with limited diagnosticity.

All relevant pieces of information (prior probabilities, conditional probabilities, updating procedure) are represented graphically and qualitatively, i.e., without numerical representations or formulas, within the hypothegon on a computer screen (Figure 3). Thus, the updating of an initial judgment does not rely on mathematical procedures. As preservice teachers are not assumed to be familiar with this representation, they first received an oral step-by-step instruction (about 20 min) that included showing the different features of the diagnostic environment. The instruction provided information about the misconceptions and the diagnostic tasks (including the sensitivity) and explained the meanings of the hypothegon, i.e., the triangle, the bar charts, and the positions of the judgment point. We also informed that we did not include students who fully understand decimals and solve comparison tasks correctly. A comprehension test with three items tested the understanding of the representation.

After the instruction, the participants had to judge 12 cases by moving the point and thus updating the probabilities for the three hypotheses. Each case represented a student (by a gender-neutral name), a task and the students’ response (with a reminder if the response was right or wrong). The prior probabilities were set to 60% for WN, 30% for ID, and 10% for SL in all cases for two reasons: First, these percentages fit to the frequencies found in studies with different age groups (see section “Decimal Strategies and Their Diagnostics”). Second, these percentages allow to differentiate between different update strategies.

As our pilot studies showed that participants need several cases to get used to the representation and stabilize their updating strategy, we implemented two analogous sequences of six task-response combinations and only analyzed the updating strategy of the second sequence. The cases were balanced with respect to the pattern of misconception–task–response combination (see Table 5): Three task responses had a high likelihood only for one misconception; three task responses had high likelihoods for two misconceptions.

**TABLE 5:** Description of the six analyzed cases in the order of the presentation.

			Likelihood of response under the condition of misconception … (presented as bar at the respective vertex)		
	Case: task and response		*WN*	*ID*	*SL*
1	3.7 > 3.02	Right	80%	20%	80%
2	4.8 < 4.63	Wrong	80%	80%	20%
3	3.49 > 3.4	Right	80%	80%	20%
4	3.7 < 3.02	Wrong	20%	80%	20%
5	4.8 > 4.63	Right	20%	20%	80%
6	3.49 < 3.4	Wrong	20%	20%	80%

### Updating Strategies (RQ1) – A Bayesian Classification Approach

In order to assess and compare the subjects’ use of update strategies, we constructed cases with values for the probabilities (priors and likelihoods) that allow for distinguishing the subjects’ diagnostic thinking (i.e., use of information, update strategy) by evaluating the evidence on their diagnostic judgment behavior (i.e., choice of posterior probabilities via location in the hypothegon).

**FIGURE 4:**
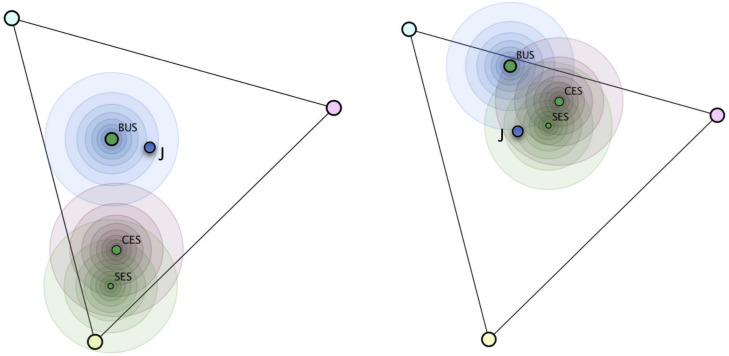
Calculated positions of the exact strategies Bayesian update strategy (BUS), combined evidence strategy (CES), single evidence strategy (SES), and a teachers’ actual judgment J for two task–response configurations. The circles define areas of similar likelihoods in 10% percentile steps. On the left hand side, judgment J has highest likelihood for BUS; on the right hand side, it can be regarded as evidence for SES, but also for CES and less for BUS.

The judgment of a subject, represented by his or her choice of position (Figure 4, left hand side) may, in some cases, be attributed unambiguously to one update strategy but may, in other cases, be consistent with more than one update strategy (Figure 4, right hand side).

In order to account for this uncertainty in interpreting a subject’s judgment, we used an analysis based on a Bayesian classification approach: We assume that each subject had a consistent update strategy and model our knowledge on the subject’s strategy by a set of probabilities:

[Fn footnote2]

$$\begin{array}{r} p_{i}\left({\hat{H}}^{\text{BUS}}\right)={\text{probability of the hypothesis}}^{\text{2}}\text{ that subject i has}\\ \text{the Bayesian update strategy (BUS),}\\ \text{and analogously }p_{i}\left({\hat{H}}^{\text{CES}}\right)\text{ and }p_{i}\left({\hat{H}}^{\text{SES}}\right) \end{array}$$

We then account for the fact that subjects only approximately determine their updated posterior in the qualitative approach, by attributing to the evidence $\hat{E}$ (i.e., the subjects’ chosen locus of a judgment) the likelihood $p\,\left(\hat{E}|{\hat{H}}^{\text{BUS}}\right)$ under the condition of him or her having a strategy (e.g., BUS) with the following Gaussian distribution

$$\begin{array}{r} p\left(\hat{E}|{\hat{H}}^{\text{BUS}}\right)=\frac{1}{N}\exp\left(-\frac{1}{d}|\hat{E}-{\hat{E}}^{\text{BUS}}{|}^{2}\right),\\ \text{and analogously }p\left(\hat{E}|{\hat{H}}^{\text{CES}}\right)\text{and }p\left(\hat{E}|{\hat{H}}^{\text{SES}}\right). \end{array}$$

$\hat{E}$ is represented by the probability vector belonging to the location of the actual judgment and ${\hat{E}}^{\mathrm{BUS}}$ by the probability vector belonging to the location when applying the BUS. Using this model to update the probability of the hypotheses *p*_*i*_ $\left({\hat{H}}^{\text{BUS}}\right)$, *p*_*i*_ $\left({\hat{H}}^{\text{CES}}\right)$, and *p*_*i*_ $\left({\hat{H}}^{\text{SES}}\right)$ on each subject with the evidence ${\hat{E}}_{i,j}$ from the cases *j* = 1…6 as described above, we define a naive Bayesian classification procedure (Duda et al., 2012). This approach has proven valid also in many cases with dependencies between the likelihoods (Domingos and Pazzani, 1997).

The normalization factor *N* of this probability density need not be calculated, since it cancels out when we evaluate *ratios* of probabilities. The parameter *d* represents the radius within the probability density decreases to $\frac{1}{\text{e}}$≈ 37% from its maximum. We chose *d* = 0.1 as a value that allows for an efficient distinction and reflects the imprecision of approximative nonnumerical judgments. For the numerical analysis of the data, we used a discrete approximation on 1,250 points in a hexagonal lattice within the hypothegon.^3^

Figure 4 illustrates the probability distribution for two cases and demonstrates how the Bayesian classification approach accounts for the fact that evidence can be considered to support more than one hypothesis on the subjects’ strategies.

When a subject judges consistently by applying one strategy in all six cases, e.g., BUS, the evidence should lead to a considerable increase in the respective posterior probability for this strategy

piposterior(H^BUS)α p(E^i,6|H^BUS)·…· p(E^i,1|H^BUS)· piprior(H^BUS)

and a decrease in the posterior probabilities for the other strategies. The classification of the subject *i* as having strategy BUS vs. CES vs. SES is then supported by the amount of change in the probability ratios. These changes of probability based on evidence are typically expressed by Bayes factors. In the present analysis, there are six possible ratios of two hypothesis, of which two are independent. BF_BUS:CES_(*i*), for example, is defined by

$$\begin{array}{c} \frac{p_{i}^{posterior}\left({\hat{H}}^{\text{BUS}}\right)}{p_{i}^{posterior}\left({\hat{H}}^{\text{CES}}\right)}=\underset{}{\underset{︸}{\prod_{j=1\ldots 6}\frac{p\left({\hat{E}}_{i,j}|{\hat{H}}^{\text{BUS}}\right)}{p\left({\hat{E}}_{i,j}|{\hat{H}}^{\text{CES}}\right)}}}\text{·}\frac{p_{i}^{prior}\left({\hat{H}}^{\text{BUS}}\right)}{p_{i}^{prior}\left({\hat{H}}^{\text{CES}}\right)}\\ {\underline{\underline{def}}}_{BF_{\text{BUS:CES }(i)}} \end{array}$$

To substantiate the classification decision for each subject, we recur to (a) the ratio of the dominant hypothesis to the subsequent one, e.g., BF_BUS:CES_(*i*) = 100:1 and (b) the highest posterior probability, when assuming equally distributed priors, e.g., $p_{i}^{p\,o\,s\,t\,e\,r\,i\,o\,r}$ $\left({\hat{H}}^{\text{BUS}}\right)$ = 99.9%.

### Influence of Instruction (RQ2)

To test our hypotheses with regard to research question 2, we designed a relevance instruction. Participants were randomly assigned to one of two conditions. Participants in the control condition did not receive further instruction and proceeded as described above. Participants in the relevance instruction condition received verbal explanations on how to incorporate all relevant information in the update following the Bayesian update strategy (without explicit reference to Bayes):

Use all the information given to you by the different bars. This works best in the following way: First, note the probabilities for the three misconceptions. Most students have the WN misconception; very few have the SL misconception. Second, look at how well each of the three misconceptions fit to the student’s response: If a student solves the problem 4.8 > 4.63 correctly, SL fits because these students are likely to solve the task correctly. Thus, the SL misconception becomes more likely. However, that does not rule out the other misconceptions: For instance, the WN misconception does not fit. Nevertheless, it is possible that the student has the WN misconception but does not answer consistently. This is quite likely because, in general, it is highly probable that a student has the WN misconception. Thus, you should consider the probabilities for the misconceptions again. (1) First, look at all the probabilities for the misconceptions. (2) Then, lock at the misconceptions that fit to the response, which ones are more likely. (3) Then, also look at the misconceptions that do not to the response, which ones are still probable [(1)–(3) was also repeated as reminder].

The instruction did not include an example of the procedure in order to avoid superficial copying of the updating strategy. In both conditions, there was a short reminder to use all information (control condition) and to remember the instruction (relevance instruction condition) just before the last six cases (i.e., before the cases chosen for the analysis).

Differences in the distribution of the three update strategies between the conditions are analyzed using a Bayesian contingency tables test (with a joint multinomial model) (Gunel and Dickey, 1974).

## Results

### Distribution of Strategies (RQ1)

The evaluation of the judgments according to the Bayesian classification approach described in section “Generating Evidence on Updating Strategies (RQ1) – The Nonnumerical Setting” resulted in a set of parameters for each participant, which allow for a classification decision:

(a) The Bayes factor BF_BUS:CES_(*i*) indicates the increase of the likelihood of one classification over the other (here, BUS over CES). Here, we focus for each subject on the ratio of the dominant hypothesis to the subsequent one, e.g., BF_1:2_(*i*) = 100:1.
(b) The posterior probabilities $p_{i}^{p\,o\,s\,t\,e\,r\,i\,o\,r}$$\left({\hat{H}}^{\text{BUS}}\right)$, $p_{i}^{p\,o\,s\,t\,e\,r\,i\,o\,r}$$\left({\hat{H}}^{\text{CES}}\right)$, and $p_{i}^{p\,o\,s\,t\,e\,r\,i\,o\,r}$ $\left({\hat{H}}^{\text{SES}}\right)$ describe the certainty of the classification under the assumption of equal priors. For example, $p_{i}^{p\,o\,s\,t,m\,a\,x}$ = 99.9% can be regarded as a 99.9% certainty of explaining a participants’ judgments by the Bayesian update strategy.

The certainty for the classification [described by both, BF_1:2_*(i)* and $p_{i}^{p\,o\,s\,t,m\,a\,x}$] of the 26 participants to one of the three assumed types of updating strategy (BUS, CES, and SES) is listed in Table 6. We indeed identified the assumed types of updating strategies known from numerical settings in our nonnumerical setting (cf. H1), with most participants classified as following either CES or SES. Only four participants were classified as using the BUS. Notably, all of these four participants were classified with very strong evidence.

**TABLE 6:** Certainty of classification.

BF_1:2_ ($p_{i}^{p\,o\,s\,t,m\,a\,x}$)	> 1 (>50.0%)	>3 (> 75.0%)	> 10 (>90.9%)	>30 (> 96.7%)	> 100 (>99.0%)	>1000 (> 99.9%)	Sum
BUS	0	0	0	1	0	3	**4**
CES	3	1	0	0	0	6	**10**
SES	1	2	2	2	1	4	**12**
**Sum**	**4**	**3**	**2**	**3**	**1**	**13**	**26**

Evidence	Weak	Moderate	Strong	Very strong	Extreme	Extreme	

Overall, most participants could be classified with strong evidence. However, four participants could only be classified with weak evidence (BF_1:2_(*i*) between 1 and 3), all of these classified as CES or SES.

### Effect of Relevance Instruction on Information Processing (RQ2)

To test whether the instruction on the relevance of priors and likelihoods (relevance instruction condition) increased the likelihood of processing more information, we compared the distribution of the three assumed strategies (BUS, CES, and SES) across the two conditions. Descriptively (see Table 7), fewer participants of the relevance instruction condition were classified as using the SES strategy in comparison to their counterparts in the control condition (cf. hypothesis 2). However, the Bayesian contingency tables test revealed a Bayes factor (BF_10_) of only 3.139. Following the interpretation of Lee and Wagenmakers (2014), a Bayes factor of 3 can be regarded as only anecdotal (or at most moderate) evidence for different distributions across the conditions.

**TABLE 7:** Distribution of strategies across conditions.

	BUS	CES	SES	Total
Control condition	1	4	9	14
Relevance instruction condition	3	6	3	12
Total	4	10	12	26

## Discussion

### Classification of Updating Strategies (RQ1)

In our study, we attempted to theoretically distinguish and empirically detect the types of updating strategies, which are suggested by the general literature on Bayesian reasoning, also in a nonnumerical setting of diagnostic judgments. As shown in Table 1, we classified these strategies with respect to different levels of information use (priors, single, or combined evidence). For most subjects in our sample, we could produce very strong evidence for their use of a BUS, CES, or SES. Overall, our results support the plausibility of the classification of strategies by the level of information use. The relatively low number of participants (4 out of 26), which included all information in their judgment and therefore can be assumed as performing (nonnumerical) Bayesian reasoning, is in line with previous findings (McDowell and Jacobs, 2017).

Notably, the only subjects (4 out of 26) with weak evidence were classified as CES or SES. This is explainable by the fact that, in our realization (i.e., with the given priors and likelihoods), these two strategies lead to less distinct posterior probabilities (cf. Figure 4). Furthermore, our classification approach was based on the assumption of a relative stability of the strategy use by each individual (cf. Cohen and Staub, 2015). It therefore does not allow to investigate any intra-individual variation of the strategy use in a similar approach as Cohen and Staub (2015).

In our study, we used a specific nonnumerical, graphical, and computer-based realization for assessment of reasoning strategies, applying a triangular representation of a ternary hypothesis space, the “hypothegon.” We consider our findings as indicative of the feasibility of this approach and envision to use the “hypothegon” paradigm for further investigations of nonnumerical reasoning (see section “Overall Discussion”).

Admittedly, there are limitations connected to the concrete realization: The approach requires a theoretically justified selection of hypothesis prior to the analysis. We chose three fundamental strategy types (BUS, CES, and SES). However, we cannot exclude that other, quite different strategy types – or mixtures of strategies – may be found to explain the subjects’ behavior. This could be investigated by further validation studies recurring either to think aloud data or to experimental variation.

Our classification of the strategies draws on a naive Bayesian classifier procedure, which allowed to rationally deal with the multiple evidence (on subjects’ judgments on different cases) and the relative contributions of each evidence to multiple hypotheses (on subjects’ possible updating strategies).

However, the robustness of the results with respect to the assumptions of this classification procedure should be reflected. We checked that a variation of the “gaussian classification radius” (*d* = 0.1) within reasonable limits (0.05 < *d* < 0.20) had no essential influence on the classification results. Furthermore, the assumption of independence of the consecutive judgments, which is essential to naive Bayesian classification, was not empirically tested within our framework but made theoretically plausible by varying and balancing the cases.

### Impact of Relevance Instruction on Information Use (RQ2)

The prevalence of non-Bayesian updating strategies (22 out of 26 subjects) suggests that (although all relevant information was presented in a salient manner) not all individuals use all information. Moreover, our results showed that the instruction on the relevance of using all probabilities (priors and likelihoods) did not substantially increase the likelihood of processing more information. Our study revealed only anecdotal evidence of an increase in the BUS and a decrease in the SES in the relevance instruction condition in comparison to the control condition. To explain this finding, we consulted literature and compared our relevance instruction to the most common approaches of supporting Bayesian reasoning in numerical settings: using frequencies instead of probabilities (e.g., Zhu and Gigerenzer, 2006; Hill and Brase, 2012) and using visual representations (Böcherer-Linder and Eichler, 2017; Pfannkuch and Budgett, 2017). These approaches can also be interpreted as setting a focus on the subset of possibilities defined by new evidence (cf. Baratgin and Politzer, 2010 for a differentiation between focusing and other revision processes). The deeper analysis of these support approaches revealed that they do not only highlight the relevance of using all information (as in our relevance instruction) but also explicitly show how these pieces of information are connected. More specifically, they display the interaction (i.e., multiplication) of likelihoods and priors as follows.

**FIGURE 5:**
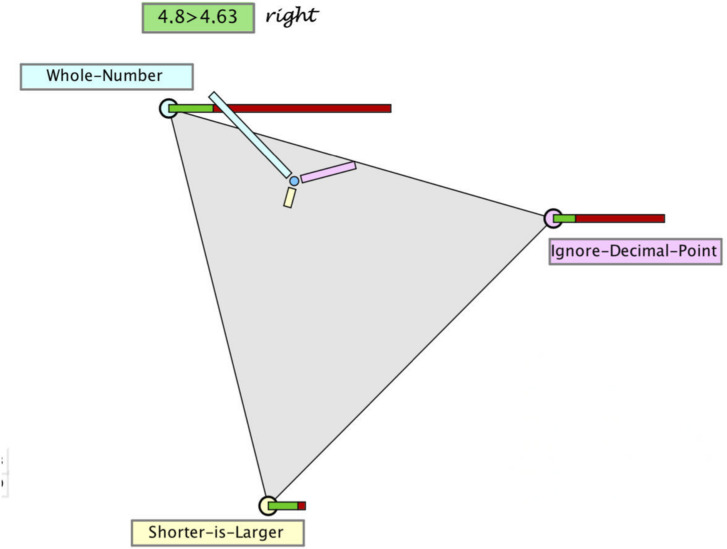
Hypothegon with visual explication of the interaction of priors and likelihoods.

If likelihoods are presented as joint frequencies (e.g., 2 of the 10 students with SL solve this task correctly), the priors (for this example 10 of 100 students) are already contained in the joint frequencies. In addition, joint frequencies verbally highlight the interaction of the likelihoods and priors (i.e., 2 of 10 of 100, called nested-set relations, Sloman et al., 2003) and thereby facilitate the construction of an adequate situation model of the prior–likelihood interaction. Another way to increase the salience of the multiplicative prior–likelihood interaction is to provide adequate visualizations (for an overview, see Khan et al., 2015). Research has shown that complementing the numerical values with nonnumerical representations that render salience to the prior–likelihood interaction (such as the unit square, e.g., Böcherer-Linder and Eichler, 2017) support Bayesian reasoning. Against this background, we devised a visual representation of the prior–likelihood interaction in our nonnumerical setting (see Figure 5) and investigated its effect on the processing of all information in a second study. By scaling the length of the likelihood bars in relation to the size of the priors, the multiplicative nature of the prior–likelihood interaction is explicitly shown and – similar as in the unit square – allows to compare the absolute lengths of the likelihood bars as direct representations of the posteriors.

## Research Question of Study 2

The finding of the predominance of non-Bayesian updating strategies within our setting, even in the relevance instruction condition, suggests that not all individuals are able to process the interaction of the information (priors and likelihoods). In numerical settings, this can be influenced by means of representations that make the interaction explicit. Therefore, we investigate the following research question (RQ3):

Does a visual explication of the prior–likelihood interaction in the nonnumerical setting increases the processing of the information in the sense of Bayesian reasoning?

H3: We hypothesize that a visual explication of the prior–likelihood interaction in an interaction explication condition leads to an increase in the BUS and a decrease in the SES in comparison to the control condition of study 1.

## Design of Study 2

Additional 16 preservice teachers from the same cohort as study 1 participated in the study. The descriptive statistics are presented in Table 4.

To test our hypotheses with regard to research question 3, we designed a visualization that makes the interaction of the probabilities (priors and likelihoods) explicit (see Figure 5).

Participants in the interaction explication condition received the same instruction as participants in the relevance instruction condition from study 1. In addition, at the end, the visualization was explained as follows: “We can see that the smaller green portion of the bar for the WN misconception is about the same size as the larger green portion of the bar for the SL misconception. Thus, if a student solves the problem correctly, it is equally likely that he or she has the WN or the SL misconception.”

## Results of Study 2

We first analyzed the certainty for the classification [both BF_1:2_(*i*) and $p_{i}^{p\,o\,s\,t,m\,a\,x}$] of the 16 new participants to one of the three assumed types of updating strategy (BUS, CES, and SES). As shown in Table 8, all participants could be classified with strong or extreme evidence. As further support for hypothesis 1 (H1), we again identified all three assumed types of updating strategies, now with most participants classified as using the BUS.

**TABLE 8:** Certainty of classification of participants in interaction explication condition.

BF_1:2_ (*p*${}_{i}^{p\,o\,s\,t,m\,a\,x}$)	> 1 (>50.0%)	>3 (>75.0%)	> 10 (>90.9%)	>30 (>96.7%)	> 100 (>99.0%)	>1000 (>99.9%)	Sum
BUS	0	0	2	0	0	7	**9**
CES	0	0	3	0	1	2	**6**
SES	0	0	0	0	0	1	**1**
Sum	**0**	**0**	**5**	**0**	**1**	**10**	**16**

Evidence	Weak	Moderate	Strong	Very strong	Extreme	Extreme	

To test whether the explication of the prior–likelihood interaction (interaction explication condition) increased the likelihood of processing the interaction of all relevant information, we compared the interaction explication condition to the control condition (study 1) with regard to the distribution of the three assumed strategies (BUS, CES, and SES). Descriptively (see Table 9), participants of the interaction explication condition were less often classified as using the SES and more often classified as using the BUS in comparison to their counterparts in the control condition (cf. hypothesis 3). The Bayesian contingency tables test revealed a Bayes factor (BF_10_) of 327.993, which can be regarded as extreme evidence for different distributions across the conditions. The results of this study are discussed within the section “Overall Discussion.”

**TABLE 9:** Distribution of strategies across conditions.

	BUS	CES	SES	Total
Control condition	1	4	9	14
Interaction explication condition	9	6	1	16
Total	10	10	10	30

## Overall Discussion

### Identifying Update Strategies in a Nonnumerical Setting

In this work, we analyzed how people update their hypotheses based on uncertain evidence (e.g., teachers’ updating their assumptions based on students’ solutions), when they only have access to nonnumerical information. Based on the results from numerical settings, we assumed that people tend to process only part of the information on relevant probabilities, resulting in updating strategies different from Bayesian reasoning. With regard to RQ1, we showed that the three assumed updating strategies (BUS, CES, and SES), which are known from numerical settings, are indeed also identifiable within the nonnumerical setting investigated in our studies.

Moreover, in line with findings from numerical settings, most participants did not follow the BUS when no further support was given. This finding supports the notion that subjects do not process and integrate all available information. Thus, we consider these findings as a validation of an information processing account of Bayesian (or non-Bayesian) reasoning. In numerical settings, the processing of information has been effectively influenced by means of instruction or representation (e.g., Khan et al., 2015; Böcherer-Linder and Eichler, 2017). In this vein, we devised similar interventions in the nonnumerical setting and conducted two studies. In study 1, an instruction on the relevance of using all probabilities (priors and likelihoods) increased the processing of more information represented in the nonnumerical setting only weakly (RQ2).

A deeper analysis of research on Bayesian reasoning revealed that not only the *quantity* of information use is relevant but also its specific *quality*, more specifically the *interaction* (i.e., multiplication) of likelihoods and priors in the judgment process. Therefore, we supplemented the intervention by explicit instruction and representation of this interaction (similar to the representations used in numerical studies, e.g., Böcherer-Linder and Eichler, 2017). In study 2, we found very strong evidence that the visual explication of the prior–likelihood interaction led to an increase in processing the interaction of all relevant information (RQ3).

These divergent effects of the two interventions suggest that many individuals do not merely fail to process all information (possibly altered by relevance instruction) but are missing to account for the interaction of these pieces of information correctly. This issue can only be influenced by reducing the necessity to convert the information. In numerical settings, this has been done effectively by presenting the probabilities as joint frequencies that already contain the priors, which automatically highlights the structure of the task (i.e., the nested-set relations, Sloman et al., 2003). Nonnumerical settings allow providing visualizations to increase the salience of the structure of the situation. This approach has already been shown effective in supporting the calculation in numerical settings (e.g., Böcherer-Linder and Eichler, 2017) and has now also proven effective in a nonnumerical setting.

To better understand this effect and also the interplay between numerical and nonnumerical information, further research with systematic combination and variation of the type of displayed information should be conducted.

### Benefits and Limitations of the Specific Nonnumerical Setting (“Hypothegon”)

The environment to investigate Bayesian reasoning in nonnumerical settings is framed and supported by the specific choice of a graphical representation, which we dubbed “hypothegon.” It comprises the triangular representation of a ternary hypothesis space and allows for the intuitive localization of probability distributions (priors and posteriors) and their change (updating). This has proven an effective setting for the nonnumerical presentation of probability information and investigation of updating strategies.

Although the hypothegon heavily relies on the ternary situation of three hypotheses (represented in two dimensions), it can be extended in two directions: Two hypotheses can be represented along a line segment (which has already been done frequently); four and more hypotheses can be represented by multiple projections of subspaces. However, the intuitive interpretation probably is limited by the ternary case. In our specific setting, we could demonstrate that it is possible to render it sufficiently comprehensible, at least to adults (cf. Table 4, Understanding of setting).

Of course, the hypothegon can be further shaped and used in research within and beyond the context of teacher judgements. In addition, within the context of teacher judgement, there are many aspects that we excluded from our studies. For example, it is plausible that teachers do not only perceive and process one piece of evidence at a time (i.e., one task–response case), but rather integrate the information from several responses from one student in order to form a decision. In the current studies, we refrained from such multistep cases to reduce complexity. However, a better understanding of how several pieces of evidence on a student interact and how teachers process this information would allow to investigate research questions such as: How much evidence do teacher process before feeling confident in the decision (cf. Codreanu et al., 2019)? Do other teacher variables, such as his or her mindset alter the number of processed evidence (cf. Weinhuber et al., 2019)?

Furthermore, teacher judgment also relies on the context of judgment and on teachers’ knowledge and goals. While in our study with student teachers, the restriction of contextual information helped to model and identify basic strategies, a more realistic setting can be expected to have considerable influence on the information processing.

### The Ecological Rationality of (Non)Bayesian Reasoning in Diagnostic Judgments

We characterized the BUS by a complete (approximate) use of probability information and Bayesian reasoning – which from a mathematical point of view can be regarded as optimal. From this point of view, the contrasting strategies (CES and SES) are characterized by a prior neglect and thus suboptimal.

By modeling the situation in a nonnumerical way (probabilities as bars, uncertainty as prior position between hypotheses), we tried to avoid the normative framing of mathematically correct statistical reasoning, which is often applied in research in Bayesian reasoning (Mandel, 2014). However, in our experimental framework, we instructed the subjects with respect to the intended interpretation of the external representation. Thus, we did not investigate their mental reasoning processes, e.g., when accepting or discarding given base rates as priors or when interpreting the change of position as update. Therefore, we would not consider judgments, which we classify as CES or SES, categorically as non-Bayesian reasoning. Baratgin (2002) as well as Baratgin and Politzer (2010) distinguish between *focusing* and *updating*. They refer to focusing when – given that all information is known and conforming to the Bayesian rule – humans revise their probability estimation by focusing on the relevant subset of the initial probability space. They refer to *updating* when humans’ posterior probability estimation is coherent with a revision of their beliefs about the situation. While we assume focusing processes when investigating the BUS strategy, our nonnumerical setting also provides an opportunity to explore subjective belief revisions more deeply.

Furthermore, we do not assume that these strategies, when applied in the diagnostic context of teachers judging students, necessarily imply better or worse performance. There may be many reasons why also normatively deficient strategies can be regarded as cognitively successful, thus reflecting perspective of ecological rationality (Simon, 1972; Kozyreva and Hertwig, 2019). As a heuristic, SES and CES may be adapted to relevant situations. For example, teachers may use a first judgment as orientation for gaining further information on the student, e.g., by selecting more specific tasks or by eliciting verbal explanations. More generally speaking, when diagnostic judgments are integrated in complex instructions, their adequacy cannot be evaluated by their local optimality. Finally, in reality, priors (base rates) may be either much less extreme and therefore less relevant than assumed here, or the probabilities used here may even be partially known or unknown to the teacher so that a more fundamental type of uncertainty arises (Gigerenzer, 2008).

In this respect, there are still many open questions as to the status of the investigated strategies within the ecology of realistic settings. A first step of investigating such question could be the analysis of the boundary conditions of “optimality” with respect to parameters and types of heuristics.

## Data Availability Statement

The datasets generated for this study are available on request to the corresponding author.

## Ethics Statement

No ethical review and approval is required for educational studies on human participants in accordance with the local legislation and institutional requirements. The participants provided written informed consent to participate in this study.

## Author Contributions

Both authors have made an equal contribution to all parts of the research and the manuscript.

## Conflict of Interest

The authors declare that the research was conducted in the absence of any commercial or financial relationships that could be construed as a potential conflict of interest.

## Footnotes

1 Each hypothesis triple (*p*_1_, *p*_2_, *p*_3_) with *p*_1_ + *p*_2_ + *p*_3_ = 1 can be found at a unique point within the hypothegon with the sizes of each *p*_*i*_ as its relative height from the side opposite to the vertex. Mathematically, this is formalized by so-called “barycentric homogeneous coordinates” (or “convex combinations”), introduced by Möbius (1827). Beyond applications in physics, chemistry, or biology, the ternary diagram (sometimes called De Finetti diagram) is occasionally used in social science to visualize normalized triples of quantified cognitive constructs (e.g., De Finetti, 1971, 2017 for distributions of subjective probability; Susmaga and Szczêch, 2015 for interestingness measures, Jøsang, 2016 for formalized subjective logic).

2 We use the circumflex accent to avoid confusion between the subjects’ (i.e., teachers’) hypotheses *H* on students’ misconceptions with our (i.e., the researchers’) hypotheses $\hat{H}$ on the teachers’ strategies.

3 The calculations were programmed by the first author in CindyScript (Richter-Gebert and Kortenkamp, 2000; www.cinderella.org), the code can be made available by request.
